# Murine in utero exposure to simulated complex urban air pollution disturbs offspring gut maturation and microbiota during intestinal suckling-to-weaning transition in a sex-dependent manner

**DOI:** 10.1186/s12989-022-00481-y

**Published:** 2022-06-15

**Authors:** Eva Guilloteau, Patrice Coll, Zhuyi Lu, Madjid Djouina, Mathieu Cazaunau, Christophe Waxin, Antonin Bergé, Ségolène Caboche, Aline Gratien, Elie Al Marj, David Hot, Laurent Dubuquoy, David Launay, Cécile Vignal, Sophie Lanone, Mathilde Body-Malapel

**Affiliations:** 1 Univ. Lille, INSERM, CHU Lille, U1286 - INFINITE - Institute for Translational Research in Inflammation, 59000 Lille, France; 2 Université Paris Cité and Univ Paris Est Créteil, CNRS, LISA, 75013 Paris, France; 3 Univ. Paris Est Créteil, INSERM, IMRB, 94010 Créteil, France; 4 Univ. Paris Est Créteil and Université Paris Cité, CNRS, LISA, 94010 Créteil, France; 5 Univ. Lille, CNRS, INSERM, CHU Lille, Institut Pasteur de Lille, UMR2014-US41-PLBS-Plateformes Lilloises de Biologie & Santé, 59000 Lille, France

**Keywords:** Air pollution, Gestational exposure, Intestinal development, Suckling-to-weaning transition, Microbiota, Maturation, Inflammation, Sex-specific

## Abstract

**Background:**

Emerging data indicate that prenatal exposure to air pollution may lead to higher susceptibility to several non-communicable diseases. Limited research has been conducted due to difficulties in modelling realistic air pollution exposure. In this study, pregnant mice were exposed from gestational day 10–17 to an atmosphere representative of a 2017 pollution event in Beijing, China. Intestinal homeostasis and microbiota were assessed in both male and female offspring during the suckling-to-weaning transition.

**Results:**

Sex-specific differences were observed in progeny of gestationally-exposed mice. In utero exposed males exhibited decreased villus and crypt length, vacuolation abnormalities, and lower levels of tight junction protein ZO-1 in ileum. They showed an upregulation of absorptive cell markers and a downregulation of neonatal markers in colon. Cecum of in utero exposed male mice also presented a deeply unbalanced inflammatory pattern. By contrast, in utero exposed female mice displayed less severe intestinal alterations, but included dysregulated expression of *Lgr5* in colon, *Tjp1* in cecum, and *Epcam*, *Car2* and *Sis* in ileum. Moreover, exposed female mice showed dysbiosis characterized by a decreased weighted UniFrac *β*-diversity index, a higher abundance of *Bacteroidales* and *Coriobacteriales* orders, and a reduced *Firmicutes/Bacteroidetes* ratio.

**Conclusion:**

Prenatal realistic modelling of an urban air pollution event induced sex-specific precocious alterations of structural and immune intestinal development in mice.

**Supplementary Information:**

The online version contains supplementary material available at 10.1186/s12989-022-00481-y.

## Background

Air pollution has been estimated to be the single most important environmental health risk factor. It represents a greater disease burden than polluted water, soil contamination, and occupational exposures combined [1]. Air pollution causes a loss of life expectancy which rivals that of tobacco smoking [2]. It is well recognized as a major risk factor for many chronic non-communicable diseases such as cardiovascular, pulmonary and metabolic diseases [3–5]. Association studies also suggest a possible contribution of air pollution to the development of neurological illnesses such as Alzheimer’s disease [6], chronic kidney disease and renal function decline [7], liver cirrhosis [8], and autoimmune diseases. For the latter, epidemiological studies have shown a relationship between exposure to air pollution and development and progression of multiple sclerosis [9], and exacerbation of rheumatoid disease [10] and systemic lupus erythematosus [11, 12]. Furthermore, air pollution may also contribute to intestinal diseases [13, 14].

There is a growing body of evidence which indicates that there is a prenatal window of susceptibility to adverse effects of air pollution. Exposure to air pollution early in life is directly linked to the development of major cardiovascular risks, including obesity, hypertension, and metabolic disorders [15, 16]. Air pollution exposure in utero has been associated with childhood asthma and allergic diseases [17, 18]. Regarding the intestinal tract, a population-based epidemiological study has highlighted that Ox exposure (as measured by redox-weighted oxidant capacity, a measure that takes into account the oxidative potential of both ozone (O_3_) and NO_2_) during the second trimester of pregnancy is associated with inflammatory bowel disease (IBD) development [19]. In mice, PM_2.5_ exposure during gestation caused changes in the distribution and structure of gut microbiota of dams [20]. However, despite the emerging role of air pollution in intestinal pathologies, animal studies focusing on the intestinal burden induced by in utero exposure to air pollution are missing. Moreover, despite ambient air pollution consisting of both particulate matter (PM) and gaseous components including O_3_, volatile organic compounds (VOCs), carbon monoxide (CO), and nitrogen oxides (NOx), realistic experimental studies that simulate complex air pollution are lacking. In this work, our hypothesis is that, in accordance with the developmental origins of health and disease (DOHAD) concept, air pollution exposure during gestation adversely affects the programming of intestinal homeostasis, which may favor the development of non-communicable chronic diseases in adulthood. We aimed to assess the early effects of gestational exposure to complex urban air pollution on intestinal tissues. For this purpose, a more representative atmospheric model, based on a 2017 pollution event in Beijing, was generated (Additional file 1: Fig. S1) [21, 22]. Pregnant mice were exposed to simulated Beijing-like air pollution from gestational day 10 (GD10) to GD17. Control mice were exposed to filtered Beijing-like air pollution during the same period. Effects of intrauterine exposure were assessed during the suckling-to-weaning transition (at postnatal day 17), which corresponds to a critical window for both structural and immune intestinal development [23, 24]. Throughout the study, we have evaluated the effects successively in the male progeny then in the female progeny, separately. These effects were assessed on four essential components of intestinal homeostasis: structural maturation, immune maturation, barrier function, and microbiota diversity and composition. In mice, as in humans, the neonatal intestine has several features that are distinct from adults. During the suckling-to-weaning period, gut growth and maturation accelerate involving both functional and structural changes in gut epithelium [25]. Thus, we first performed an overall assessment of the gut mucosal structure. The gut structural maturation was evaluated by measuring submucosa cellularity and mucosal surface area in proximal colon, and the villus length, depth, and villus/crypt ratio in ileum. Furthermore, during the suckling-to-weaning period, in small intestine, vacuolated fetal-type epithelium is replaced by non-vacuolated adult-type epithelium [25]. Since the ileum of postnatal day17 pups presented intense vacuolation of the villi, we developed a method for quantitative assessment of this vacuolation. Then, to further examine epithelium proliferation, immunohistochemical staining of the proliferative marker PCNA (proliferating cell nuclear antigen) was performed. We analyzed the epithelium differentiation by quantifying the levels of several markers of intestinal cells: The leucine-rich-repeat-containing G-protein-coupled receptor 5 (*Lgr5*), the best described intestinal stem cell marker; the markers of absorptive cells alkaline phosphatase, intestinal (*Alpi)*, epithelial cell adhesion molecule (*Epcam),* and carbonic anhydrase 2 *(Car2);* the markers of secretory cells namely mucin 2 (*Muc2*) for goblet cells, lysozyme 1 (*Lyz1*) for Paneth cells, chromogranin A (*ChrgA*) for enterochromaffin cells and POU domain, class 2, transcription factor 3 (*Pou2f3*) for tuft cells. To better assess the functional maturation of the epithelium, we quantified the levels of certain enzymes representative of neonatal epithelium or adult epithelium. During the suckling period, neonatal intestinal cells express disaccharidase lactase-phlorizin hydrolase (*Lct*) to digest milk lactose [1]. After the suckling period, enterocytes adapt to digest solid food that is rich in complex carbohydrates and low in fat. This is manifested by a switch in brush border disaccharidase expression from lactase to sucrase isomaltase (*Sis*) and trehalase (*Treh*) [2]. Furthermore, another metabolic switch is related to the low concentration of arginine in milk. To provide for the need for arginine, neonatal enterocytes express the rate limiting enzyme in arginine biosynthesis, argininosuccinate synthase-1 (*Ass1*) [3]. In contrast, adult enterocytes express arginase 2 (*Arg2*), an enzyme capable of catabolizing arginine that is abundant in solid foods. Furthermore, the neonatal intestinal epithelium expresses the neonatal Fc fragment of the IgG receptor and transporter *(Fcgrt*, also called *FcRn*), which mediates absorption of maternal IgG from milk into the bloodstream [26]. The expression of *Fcgrt* declines significantly during the suckling-to-weaning transition [24]. Similarly, the expression of *Prdm1* (PR domain containing 1, with ZNF domain; also called *Blimp-1*, or B lymphocyte-induced maturation protein-1) is lost at the suckling-to-weaning transition [27, 28].

The assessment of gut immune maturation was based on quantification of transcription factors and inflammatory cytokines representative of the Th1, Th2, Th17, and Treg immune response. For gut barrier function, we measured the levels of 3 markers of intestinal permeability, occludin (Ocln), tight junction protein-1 (Tjp1; coding for zonula occludens 1 protein), and claudin-4 (Cldn4). Finally,

## Results

Gestational exposure to Beijing -like air did not change litter size (Additional file 1: Fig. S2A, *p* = 0.9), offspring sex distribution (Additional file 1: Fig. S2B, *p* = 0.3, Fisher’s exact test), dam body weight (Additional file 1: Fig. S2C, *p* = 0.5). Notably, body weight of offspring at termination PND17 were similar between control air and Beijing-like air exposed pups (Additional file 1: Fig. S2D, *p* = 0.3).

### Histomorphological analysis in male mice

**Fig. 1:**
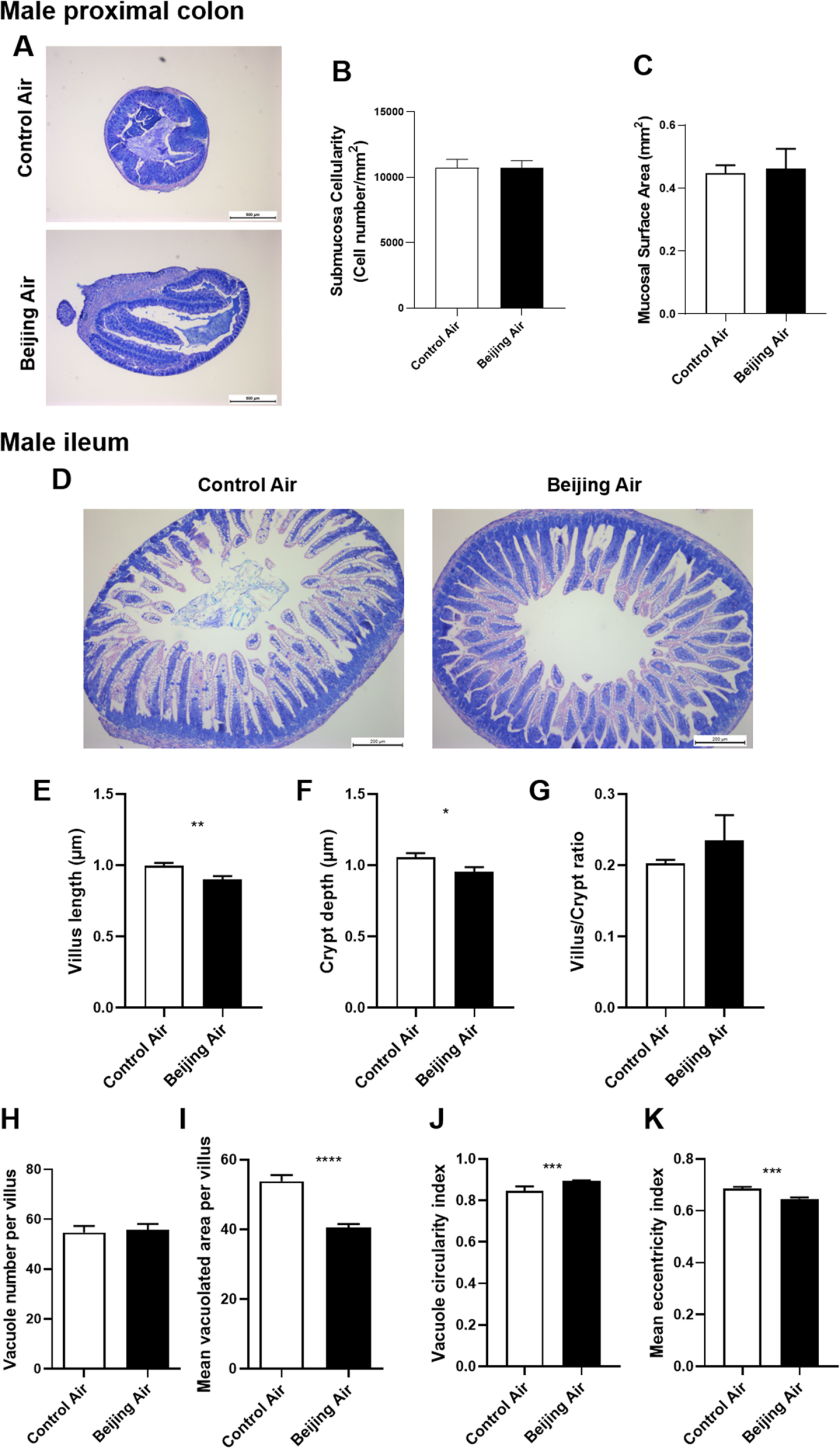
Histomorphological analysis in male mice. **A** Representative pictures of MGG-stained proximal colon from control air- and Beijing-like air-exposed mice, scale bar 500 µm (n = 10/group). **B** Submucosa cellularity. **C** Mucosal surface area. **D** Representative pictures of MGG-stained ileum from control air- and Beijing-like air-exposed mice, scale bar 200 µm (n = 10/group). **E** Villus length. **F** Crypt depth. **G** Villus/crypt ratio. **H** Vacuole number per villus. **I** Mean vacuolated area per villus. **J** Vacuole circularity index. **K** Mean eccentricity index. *p < 0.05, **p < 0.01, ***p < 0.005, ****p < 0.001 as determined by the Mann–Whitney U test

As a first assessment of gut mucosal structure, May Grünwald Giemsa (MGG)-stained sections of male colon and ileum were analyzed (Fig. 1A, D). In colon, the cell number in the submucosa and the mucosal surface area were measured (Fig. 1B, C). No variation was evident. In ileum, the villus length and the crypt depth were measured (Fig. 1E, F). They were both significantly decreased in Beijing-like air exposed mice compared to control air mice. The villus/crypt ratio did not vary (Fig. 1G). The number of vacuoles per villi was determined and did not show variation between the 2 groups of mice (Fig. 1H). The mean vacuolated area per villus was significantly reduced in Beijing-like air exposed mice (Fig. 1I). Morphometric evaluation of the vacuoles also showed significant variations (Fig. 1J, K). In pups exposed in utero to air pollution, the vacuole circularity index was increased and the mean eccentricity index was decreased compared to control pups. Therefore, in males, postnatal exposure to air pollution induced histomorphological alterations in ileum, and notable disturbances of the vacuolation process.

### Histomorphological analysis in female mice

**Fig. 2:**
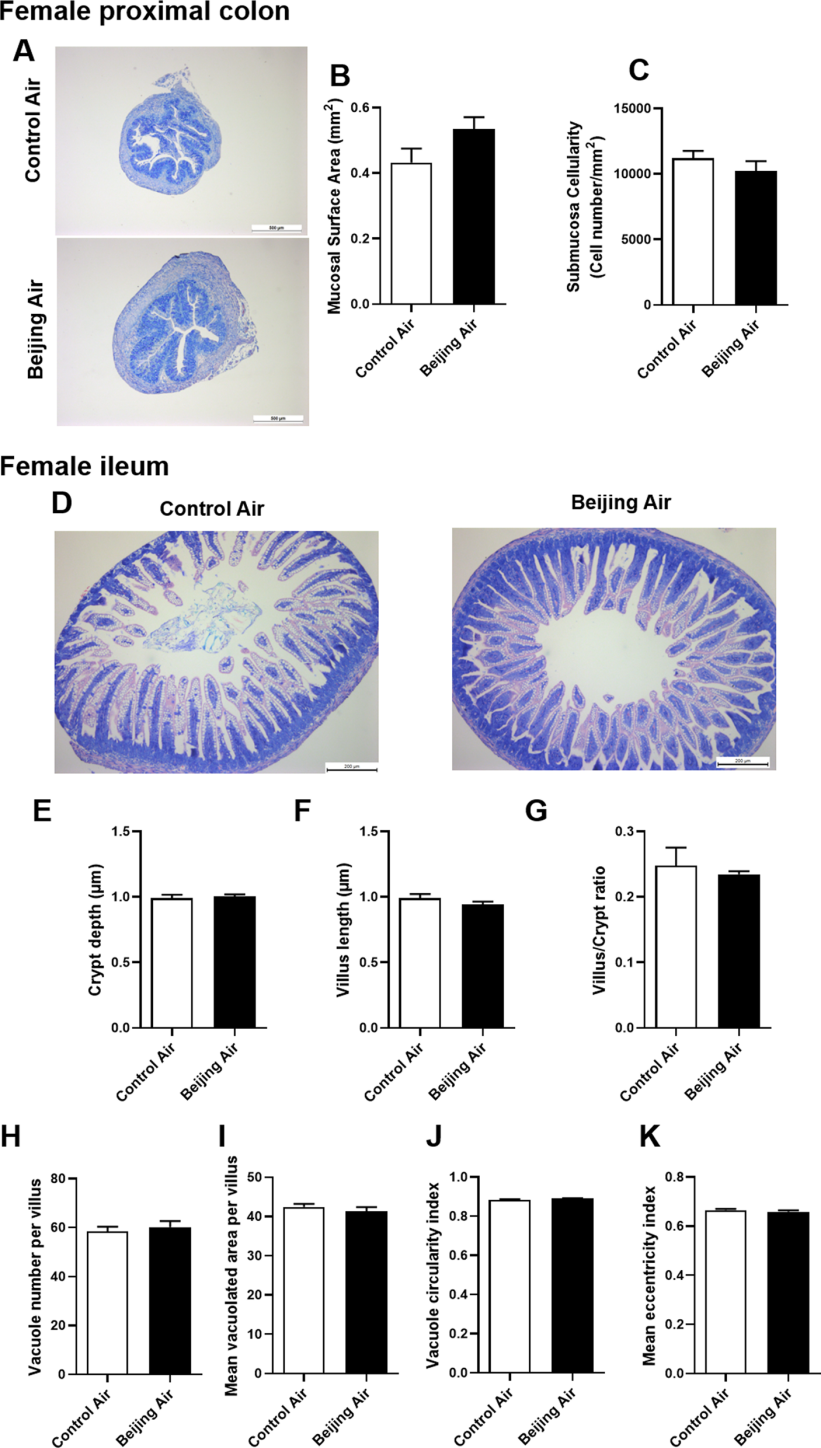
Histomorphological analysis in female mice. **A** Representative pictures of MGG-stained proximal colon from control air- and Beijing-like air-exposed mice, scale bar 500 µm (n = 10/group). **B** Submucosa cellularity. **C** Mucosal surface area. **D** Representative pictures of MGG-stained ileum control air- and Beijing-like air-exposed mice, scale bar 200 µm (n = 10/group). **E** Villus length. **F** Crypt depth. **G** Villus/crypt ratio. **H** Vacuole number per villus. **I** Mean vacuolated area per villus. **J** Vacuole circularity index. **K** Mean eccentricity index

We performed the same analyses in colon and ileum of female pups. In colon, the mucosal surface area and the submucosa cellularity did not show significant variation between air pollution- and control-exposed mice (Fig. 2A–C). In ileum, there were no modification of the crypt depth, villus length, and villus/crypt ratio (Fig. 2D–G). The morphometric analyses of the vacuoles showed no differences between the female pups in utero exposed to simulated Beijing-like polluted air compared to female control pups. Therefore, in females, no important histological impairments of colon and ileum were induced by the postnatal exposure of simulated Beijing-like air pollution.

### Epithelium proliferation, differentiation, and maturation in male mice

**Fig. 3:**
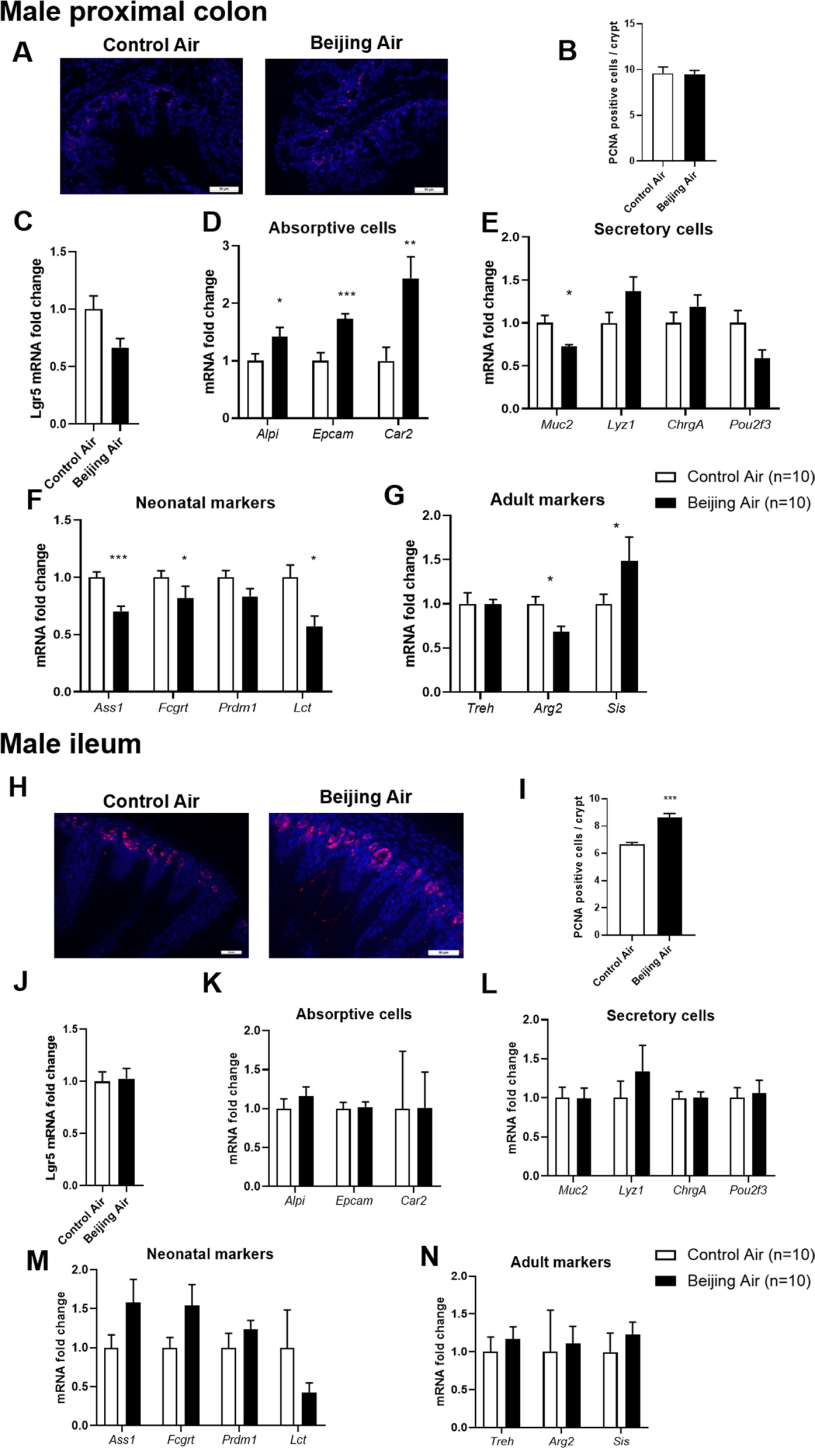
Epithelium proliferation, differentiation, and maturation in male mice. **A** Representative pictures of PCNA-stained proximal colon from control air- and Beijing-like air-exposed mice, scale bar 50 µm (n = 10/group). **B** Number of PCNA positive cells/crypt. **C** *Lgr5* transcript levels. **D** *Alpi, Epcam,* and *Car2* transcript levels. **E** *Muc2, Lyz1, ChrgA*, and *Pou2f3* transcript levels. **F** *Ass1, Fcgrt, Prdm1, and Lct* transcript levels*.* **G** *Treh, Arg2,* and *Sis* transcript levels. **H** Representative pictures of PCNA-stained ileum from control air- and Beijing-like air-exposed mice, scale bar 50 µm (n = 10/group). **I** Number of PCNA-positive cells/crypt. **J** *Lgr5* transcript levels. **K** *Alpi, Epcam,* and *Car2* transcript levels. **L** *Muc2, Lyz1, ChrgA,* and *Pou2f3* transcript levels. **M** *Ass1, Fcgrt, Prdm1,* and *Lct* transcript levels. **N** *Treh, Arg2,* and *Sis* transcript levels. *p < 0.05, **p < 0.01, ***p < 0.005 as determined by the Mann–Whitney U test

In male colon, the number of cells positive for the proliferative marker PCNA was counted and was similar between the polluted air- and the control air-exposed mice (Fig. 3A, B). Transcript levels of several markers of intestinal cells were quantified by real-time polymerase chain reaction (PCR).The levels of the intestinal stem cell marker *Lgr5* did not show significant variation (Fig. 3C).The 3 markers of absorptive cells, *Alpi*, *Epcam* and *Car2* were all significantly upregulated in Beijing-like air exposed mice compared to control air mice (Fig. 3D). The marker of goblet cells mucin 2 (*Muc2*) was significantly downregulated in Beijing-like air exposed mice (Fig. 3E). The transcript levels of the markers of Paneth cells, *Lyz1*, of enterochromaffin cells, *ChrgA*, and of tuft cells *Pou2f3*, were not changed between the 2 groups of mice.

To assess the functional maturation of gut epithelium, we quantified mRNA levels of several neonatal and adult markers in colon of Beijing-like air exposed mice compared to control air mice (Fig. 3F, G) [24]. In accordance with the physiological metabolic switch occurring after the suckling period in order to digest solid food instead of milk, expression of *Lct* was downregulated, and expression of *Sis* was upregulated; however, the expression of *Treh* was unchanged. Colon expression of *Ass1* and *Arg2* were lower in mice exposed in utero to Beijing-like air pollution as compared with mice exposed to control air pollution. Neonatal marker *Prdm1* expression was not modified but the one of *Fcgrt* was significantly reduced in colon after Beijing-like air pollution exposure.

In ileum, the number of PCNA-positive cells was higher in Beijing-like air exposed mice compared to control air mice, reflecting an increase of epithelial proliferation in these mice (Fig. 3H, I). The mRNA levels of the markers of stem, absorptive, and secretory cells were not modified (Fig. 3J–L). Moreover, there was no variation of expression of the neonatal and adult genes (Fig. 3M, N).

Altogether, these results tend to show that postnatal exposure to simulated Beijing-like air pollution in male pups led to alterations of differentiation and maturation biomarkers in colon epithelial cells, as well as to an increase of epithelial proliferation in ileum.

### Epithelium proliferation, differentiation, and maturation in female mice

**Fig. 4:**
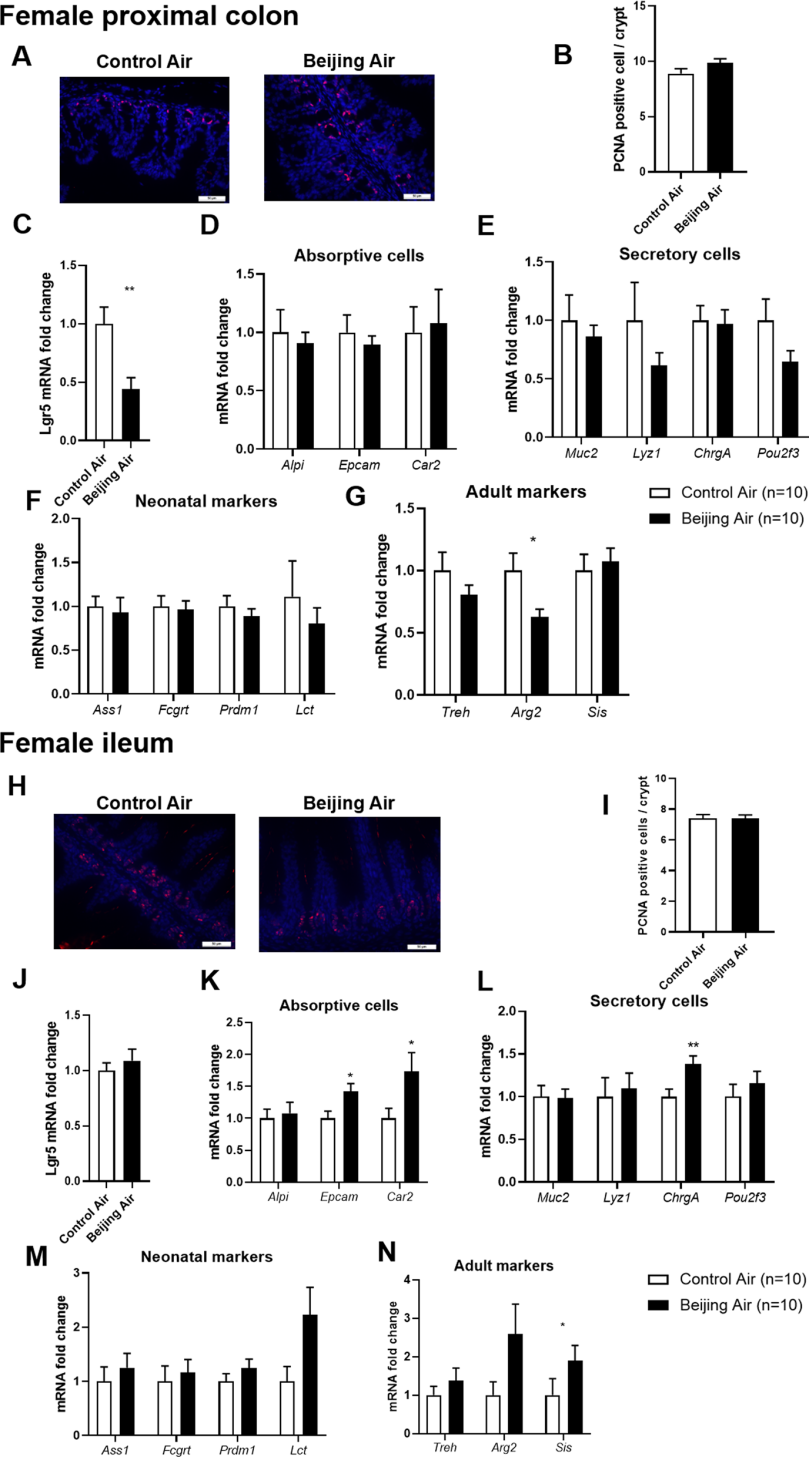
Epithelium proliferation, differentiation, and maturation in female mice. **A** Representative pictures of PCNA-stained proximal colon from control air- and Beijing-like air-exposed mice, scale bar 50 µm (n = 10/group). **B** Number of PCNA-positive cells/crypt. **C** *Lgr5* transcript levels. **D** *Alpi, Epcam*, and *Car2* transcript levels. **E** *Muc2, Lyz1, ChrgA*, and *Pou2f3* transcript levels. **F** *Ass1, Fcgrt, Prdm1,* and *Lct* transcript levels. **G** *Treh, Arg2,* and *Sis* transcript levels. **H** Representative pictures of PCNA-stained ileum from control air- and Beijing-like air-exposed mice, scale bar 50 µm (n = 10/group). **I** Number of PCNA-positive cells/crypt. **J** *Lgr5* transcript levels. **K** *Alpi, Epcam,* and *Car2* transcript levels. **L** *Muc2, Lyz1, ChrgA,* and *Pou2f3* transcript levels. **M** *Ass1, Fcgrt, Prdm1*, and *Lct* transcript levels. **N** *Treh, Arg2,* and *Sis* transcript levels. *p < 0.05, **p < 0.01 as determined by the Mann–Whitney U test

In females, PCNA immunostaining of colon showed no significant modification of proliferation in Beijing-like air exposed mice compared to control air mice (Fig. 4A, B). The mRNA level of *Lgr5* was significantly downregulated in Beijing-like air exposed mice (Fig. 4C). The transcript abundance of absorptive and secretory cell markers did not show significant variation (Fig. 4D, E). The neonatal markers were not changed either (Fig. 4F). The transcription of *Arg2* was significantly downregulated in Beijing-like air exposed mice compared to control air mice (Fig. 4G).

In ileum, PCNA staining was similar between the Beijing-like air exposed mice and the control air mice (Fig. 4H, I). The level of *Lgr5* was also unchanged (Fig. 4J). The expression of absorptive cell markers *Epcam* and *Car2* were upregulated in Beijing-like air exposed mice (Fig. 4K). Among the secretory cell markers, a relative over-expression of *ChrgA* transcripts was quantified in Beijing-like air exposed mice as compared with control air mice (Fig. 4L). The maturation of ileal tissue was weakly altered, and an upregulation of *Sis* expression was detected in Beijing-like air exposed mice (Fig. 4M, N). Because over-expression of *ChgrA* in Beijing-like air-exposed mice ileum was observed, we quantified expression of other markers of enteroendocrine cells. Levels of tachykinin 1 (*Tac1*, or substance P)-producing enterochromaffin cells, glucagon (*Gcg*)- and peptide YY (*Pyy*)-expressing L cells, gastric inhibitory protein (*Gip*)-producing K cells, neurotensin (*Nts*)- and neuromedin-producing N cells, and secretin (*Sct*)-producing S cells were quantified [29] (Additional file 1: Fig. S3). An upregulation of *Nts* was found in the Beijing-like air-polluted group, reflecting an abnormal expression of neurotensin.

### Inflammatory pattern in male mice

**Fig. 5:**
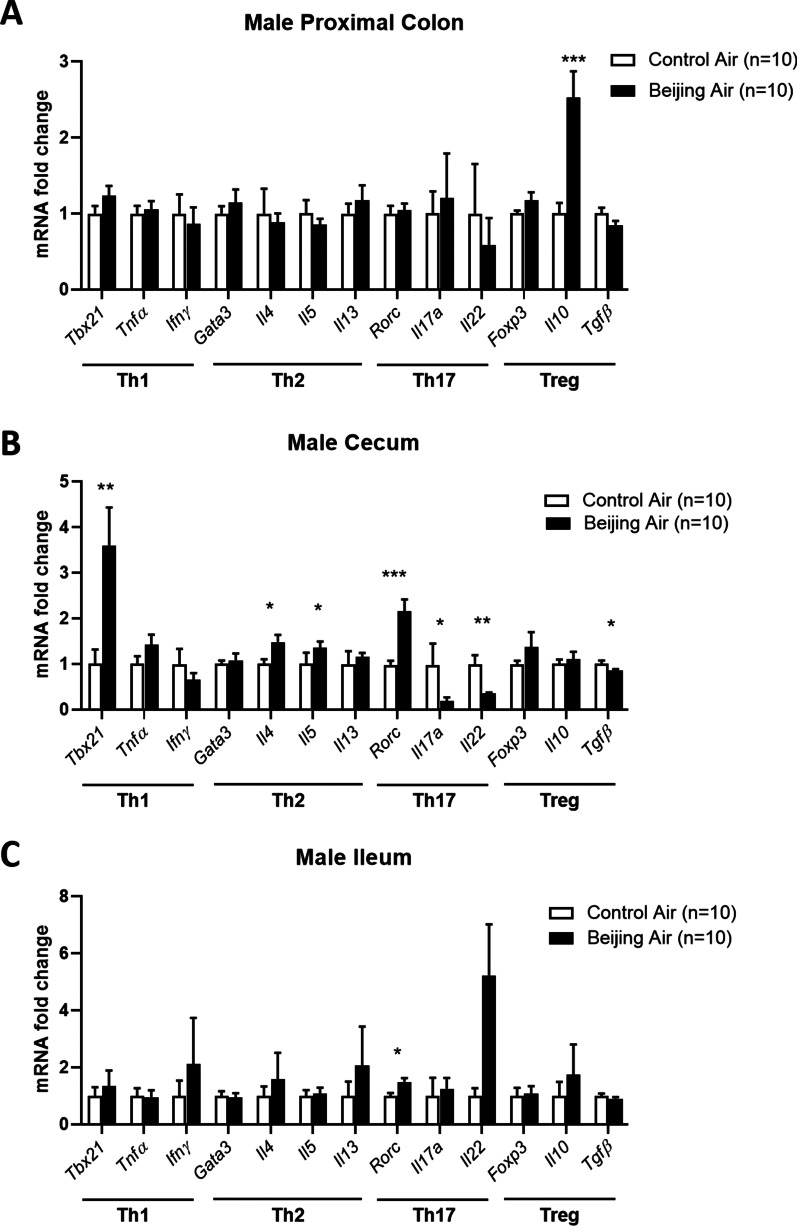
Inflammatory pattern in male mice. Transcript levels of Th1 (*Tbx21, Tnf*α*, Ifn*γ), Th2 (*Gata3, Il4, Il5, Il13*), Th17 (*Rorc, Il17a, Il22*), and Treg (*Foxp3, Il10, Tgfβ*) genes in proximal colon (**A**), cecum (**B**), and ileum (**C**) from control air- and Beijing-like air-exposed male mice (n = 10/group). *p < 0.05, **p < 0.01, ***p < 0.005 as determined by the Mann–Whitney U test

In order to assess whether in utero exposure to air pollution induces impairments of the inflammatory process in postnatal day 17 pups, we quantified mRNA levels of transcription factors and inflammatory cytokines representative of the Th1, Th2, Th17, and Treg immune response. In proximal colon, expression of the immunomodulatory cytokine *Il10* was strongly enhanced in Beijing-like air pups compared to control air pups (Fig. 5A). In cecum, the Th1 transcription factor *Tbx21* (also called *T-bet*) was significantly higher in Beijing-like air exposed mice compared to control mice (Fig. 5B). The Th2 cytokines *Il4* and *Il5* were also upregulated in Beijing-like air pups. The Th17 transcription factor *Rorc* was upregulated and the levels of *Il17a* and *Il22* were downregulated in Beijing-like air exposed mice compared to control air mice. Levels of *Tgfβ* were also lower in cecum of Beijing-like air exposed mice. In ileum, a significant upregulation of *Rorc* was induced by in utero exposure of simulated Beijing-like air pollution (Fig. 5C).

### Inflammatory pattern in female mice

**Fig. 6:**
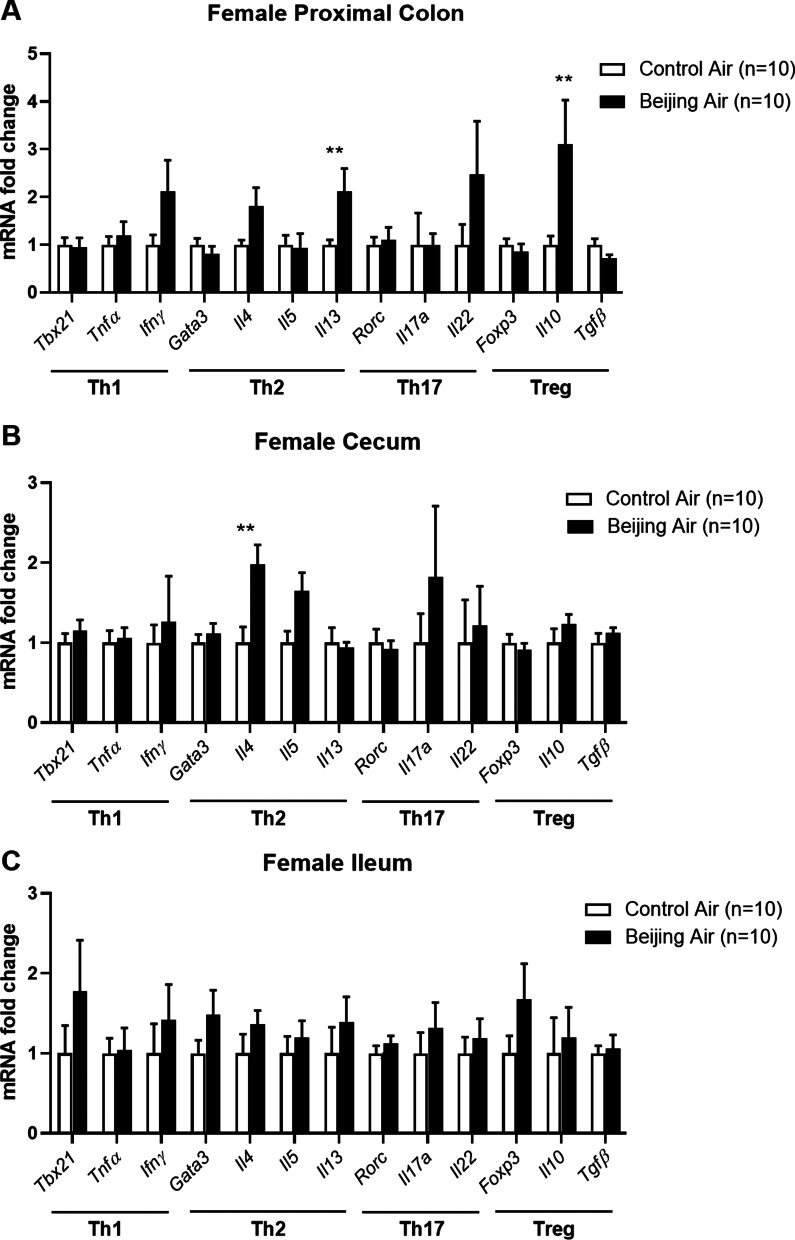
Inflammatory pattern in female mice. Transcript levels of Th1 (*Tbx21, Tnf*α*, Ifn*γ), Th2 (*Gata3, Il4, Il5, Il13*), Th17 (*Rorc, Il17a, Il22*), and Treg (*Foxp3, Il10, Tgfβ*) genes in proximal colon (**A**), cecum (**B**), and ileum (**C**) from control air- and Beijing-like air-exposed male mice (n = 10/group). **p < 0.01 as determined by the Mann–Whitney U test

In proximal colon of female mice, the transcript levels of *Il13* and *Il10* cytokines were greatly upregulated in Beijing-like air exposed mice compared to control air mice (Fig. 6A). In addition, the mRNA expression of *Il4* was enhanced in cecum by in utero Beijing-like air exposure (Fig. 6B). None of the other markers studied were modified in ileum (Fig. 6C).

### Intestinal permeability markers in male and female mice

**Fig. 7:**
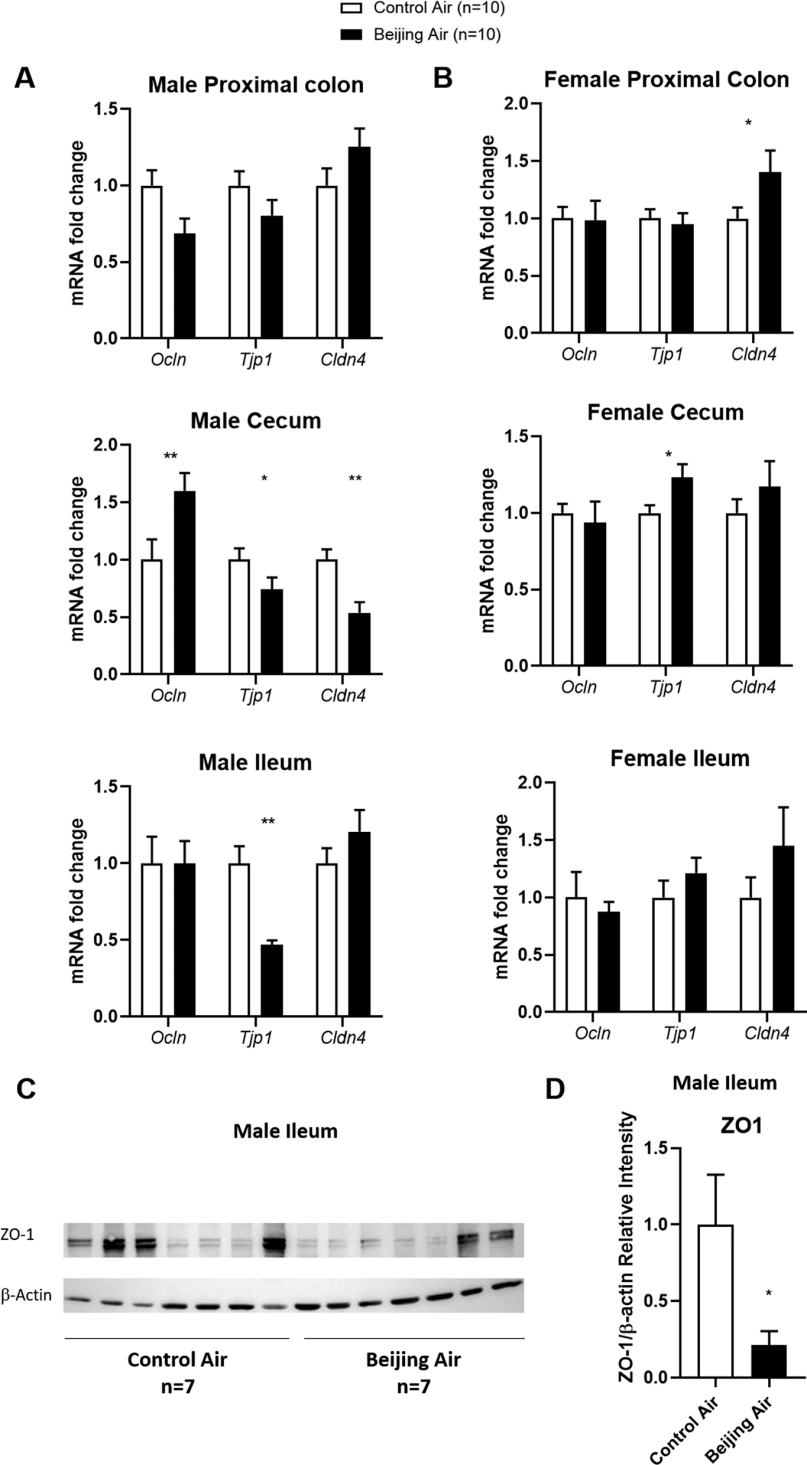
Tight junction expression in male and female mice. **A-B** Transcript levels of *Ocln*, *Tjp1* and *Cldn4* in proximal colon, cecum, and ileum of male (**A**) and female (**B**) mice exposed to control air or Beijing-like air (n = 10/group). **C** ZO-1 and *β*-actin western blot analysis of male ileum. **D** Densitometric analysis of ZO-1 western blots. *p < 0.05, **p < 0.01 as determined by the Mann–Whitney U test

We assessed whether in utero exposure to air pollution disrupts the intestinal barrier. In male proximal colon there was no significant expression variation of the 3 markers of intestinal permeability, *Ocln*, *Tjp1*, and *Cldn4* (Fig. 7A). In cecum of Beijing-like air-exposed males, we observed an upregulation of *Ocln* and a downregulation of *Tjp1* and *Cldn4* transcripts. *Tjp1* expression was also strongly reduced in the ileum of males exposed to Beijing-like air. In females, in utero Beijing-like air pollution exposure induced an upregulation of *Cldn4* and *Tjp1* in proximal colon and cecum, respectively (Fig. 7B). Furthermore, in order to confirm the relevancy of the strong decrease of Tjp1 transcript expression in male ileum, we quantified the expression of ZO-1 protein by western blot and confirmed that ZO-1 protein levels were significantly reduced in ileum of male mice exposed in utero to Beijing-like air pollution compared to control air mice (Fig. 7C, D).

### Microbiota DNA extraction and 16S rDNA gene amplicon sequencing analysis

**Fig. 8:**
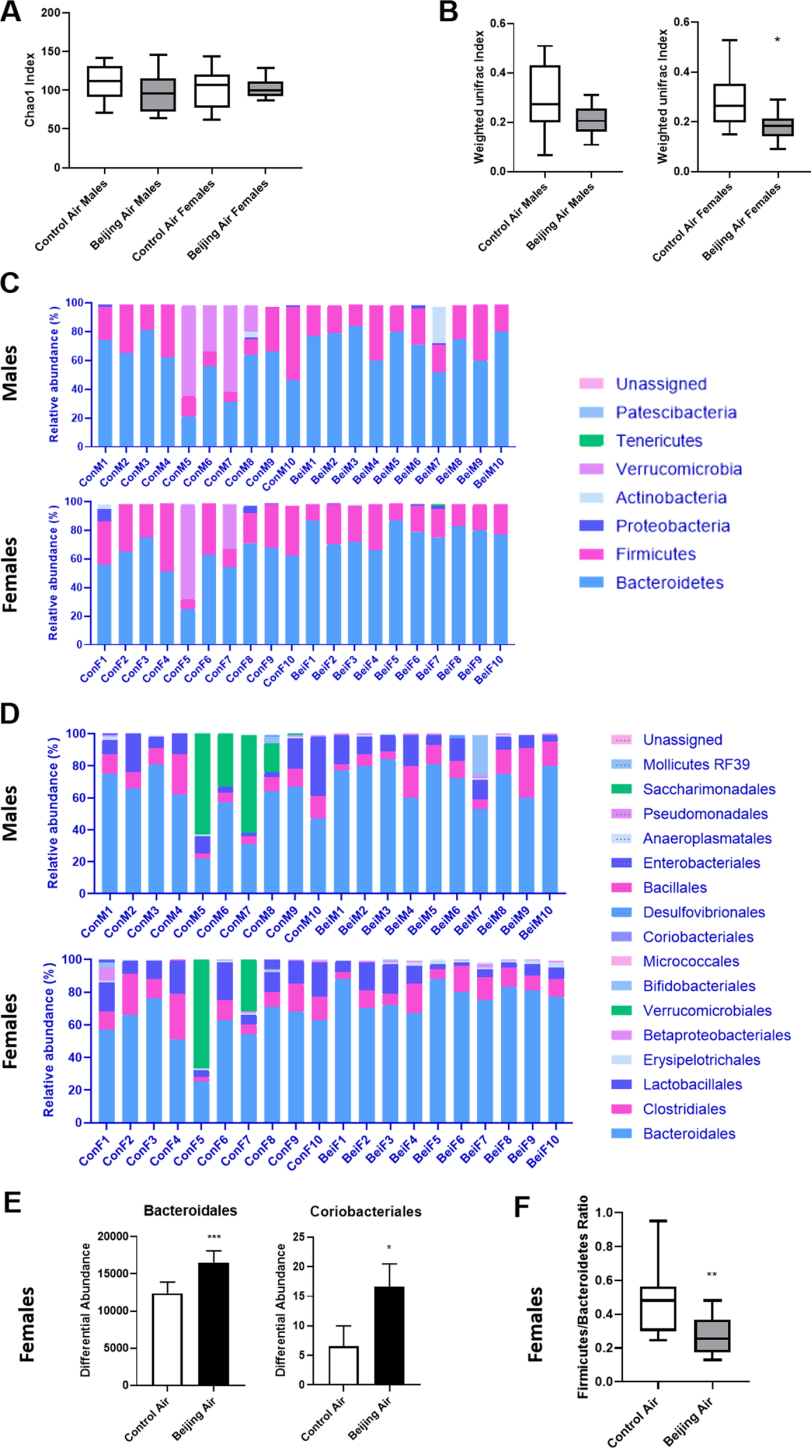
Luminal content microbiota analysis in male and female mice exposed in utero to control air or Beijing-like air (n = 10/group). **A** Chao1 α-diversity index. **B** Weighted UniFrac *β*-diversity index. **C** Overview of the relative abundance of gut bacteria depicted at the phylum level. **D** Overview of the relative abundance of gut bacteria depicted at the order level. **E** The differential abundance of significantly changed bacterial orders in female mice. *p < 0.05, ***p < 0.005 as determined by Corncob. **F** *Firmicutes/Bacteroidetes* ratio. **p < 0.01 as determined by the Mann–Whitney U test

To assess the impact of in utero exposure to air pollution on colon luminal bacterial content, we sequenced V3-V4 amplicons of 16S rRNA genes. After a denoising step performed with DADA2 software, we obtained a total of 884,707 reads. Exposure to air pollution did not significantly affect α-diversity (Chao1 diversity index, Fig. 8A; Evenness and Simpson indices, Additional file 1: Fig. S4). The weighted UniFrac *β*-diversity index showed no significant difference in males, but a significant decrease in females (*p* = 0.011; Fig. 8B). After taxonomic assignment of amplicon sequence variants (ASVs), the effect of in utero air pollution exposure on the abundance of phyla was assessed. Taxonomic assignment at the phylum level of ASVs, with each color representing an individual bacterial phylum, is shown in Fig. 8C. Bacterial composition was dominated by members of the *Bacteroidetes* phylum followed by *Firmicutes*, although mice presented important interindividual variations. A high abundance of *Verrucomicrobia* was found in only 4 control male and 2 control female mice, but no phylum was found to be significantly different between the exposed and control mice. Similarly, analyses at the class level showed important interindividual variability, but did not reveal significant variations between air pollution- and control-exposed groups (data not shown). At the order level, the main bacteria were *Bacteroidales, Clostridiales,* and *Lactobacillales* (Fig. 8D). In males, no bacterial order was found to vary significantly following air pollution gestational exposure. By contrast, *Bacteroidales* and *Coriobacteriales* orders were significantly more abundant in Beijing-like air-exposed mice than in control air-exposed female mice (*p* = 0.001 and *p* = 0.04, respectively; Fig. 8E). Moreover, the *Firmicutes*/*Bacteroides* ratio, which is a widely used marker of intestinal dysbiosis, was calculated and found to be significantly reduced in females after in utero exposure to Beijing-like air (Fig. 8F).

## Discussion

Although exposure to air pollution during pregnancy is linked to high risk of adverse pregnancy outcomes and long-term postnatal health, limited mechanistic data exists to assess these impacts under controlled exposure conditions. This limitation of our knowledge is mainly attributed to the complexity of the polluted atmosphere, and to the great difficulty in modelling the impact of realistic exposures. Using CESAM, an atmospheric simulation chamber, we have capitalized on a totally innovative platform for exposing mice to more realistic atmospheric conditions. Among the constituents of air pollution associated with deleterious effects on health, we considered gaseous pollutants (O_3_, SO_2_, CO, NOx, and VOCs) and particles (PM_10_, PM_2.5_, and ultrafine PM). In order to simulate atmospheric mixtures in all their complexity in the laboratory, environmental chemists have developed photo-reactors that are equipped to reproduce and control atmospheric processes such as solar radiation, concentrations of species, and the timely injection of aerosols and gases. These atmospheric simulation chambers thus offer the possibility of studying the myriad of products resulting from the atmospheric oxidation of primary compounds [30].

This innovative experimental approach allowed us to mimic the effects of “real life” exposure of urban air pollution on the suckling-to-weaning intestinal tissues. In mice intestinal development begins right before birth and intestinal maturation is completed at approximately 3 weeks postnatally during the suckling-to-weaning transition [31]. In female offspring, in utero exposure to Beijing-like air pollution induced mild modifications of intestinal development. Enhanced expression of *Epcam* and *Car2* absorptive epithelial cell markers and *ChrgA* enteroendocrine cell marker were observed in small intestine. In colon, the only alterations were decreased expression of *Lgr5* stem cell marker and *Arg2* adult epithelial cell marker. Therefore, our results argue in favor of weak effects of postnatal air pollution exposure on intestinal differentiation and maturation in female young offspring. By contrast, in males, several parameters of intestinal development were affected by air pollution exposure. In colon, the 4 neonatal markers studied were found downregulated in Beijing air exposed mice, but less consistency was observed for the adult markers. It is likely that the development of some epithelial functions is disturbed in male colon, and further studies should be conducted in order to identify them. Besides, in male small intestine, both the villus length and the crypt depth were reduced. This was associated with increased epithelial proliferation, as well as decreased size and morphological alterations of the vacuoles. This latter observation may reflect a trend to replacement of vacuolated fetal-type epithelium with non-vacuolated adult-type epithelium and therefore more precocious maturation in in utero pollution-exposed mice. However, the markers of neonatal cells were not changed, indicating the absence of a functional impact of exposure to air pollution on intestinal maturation on the parameters studied. Therefore, these results suggest that in males, in utero exposure to air pollution would promote only partially some physiological processes of small intestinal maturation. Moreover, our results show that in utero exposure to Beijing-like air pollution led to disturbances of proliferation processes. It is known that villus and crypt morphogenesis occurs during embryogenesis and postnatally, respectively; crypts are the architectural unit of the stem cell niche [32]. Villus and crypt morphogenesis are complex processes which are essential for normal intestinal physiology. A precedent for imprinting of skin epithelial stem cells has been reported [33]. Therefore, the abnormalities observed in male small intestine may reflect disorders of intestinal development which deserve further study.

We also studied whether in utero air pollution exposure could affect the maturation of the immune system. The expression of the immunomodulatory cytokine IL-10 was exacerbated in colon by in utero exposure to Beijing-like air, both in males and females. In neonates, as in adults, IL-10 can have anti-inflammatory properties and can be produced by macrophages and T cells [34]. IL-10R signaling in macrophages is pivotal in confining a microbiota-driven inflammatory response beginning at the third week of life [35]. IL-10 can also be produced by neonatal type 1 conventional dendritic cells (cDC1) before their differentiation into IL-12p40-producing cDC1 [36]. Therefore, if IL-10 overexpression derives from cDC1, it could reflect a defect in maturation of these cells. Apart from this finding, the cytokine profile in the colon was not altered. In ileum, the cytokine pattern was very similar between Beijing-like air exposed mice and control air exposed mice. By contrast, in cecum, and only in males, we observed an increase of 2 major immune transcription factors (*Tbx21* and *Rorc*), an overexpression of Th2 cytokines (*Il4* and *Il5*), and decreased expression of cytokines *Il17a*, *Il22*, and *Tgfβ*. These alterations are numerous but they do not make it possible to demonstrate a clear defect in the immune response. Association of the upregulation of Th17 transcription factor *Rorc* and downregulation of Th17 cytokines *Il17a* and *Il22* is paradoxical, and to date the data in the literature is not sufficient to fully explain this apparent discrepancy. It is known that immune homeostasis in the gut is normally maintained by the production of low levels of IL-17A and IL-22 by resident Th17 lymphocytes [37] and ILC3 [38]. In neonates IL-17A can also be produced by γδT cells and has an essential role in host defense against *C.difficile* infection [39].

Another important parameter of intestinal immune response is the development of an efficient mucosal barrier including a tailored regulation of tight junctions which seal the epithelial cell–cell contacts and regulate the paracellular passage of solutes [40]. The modifications of permeability markers that we observed, particularly downregulation of ZO-1 at the transcript and protein levels in male ileum, could lead to a gut barrier defect. The leakiness of the gut epithelium has been associated with the development of allergic and autoimmune diseases, especially when it is associated with a dysbiotic microbiota that cross the damaged barrier [41].

From birth, the normal gut microbiota contributes to the development of gut function, educates the immune system, contributes to the regulation and maintenance of intestinal barrier function, provides protection against infection, and promotes tolerance of foods. In addition, the early life microbiota has a crucial role in the risk of acquiring diseases such as asthma, atopic dermatitis, diabetes, allergic diseases, obesity, cardiovascular diseases, and neurological disorders [42]. In our study, in utero exposure to air pollution did not modify the α-diversity (within sample diversity) and especially the Chao1 species richness index, indicating that the number of different species was similar between the groups. By contrast, and only in females, the bacterial composition showed few significant variations. At the phylum level, the ratio of *Firmicutes/Bacteroidetes* was reduced. In adults, this ratio is a hallmark of low-grade inflammation [43]. It has also been proposed as a marker of intestinal maturation during aging (in the second year of adulthood) [44]. In female mice exposed in utero to air pollution, the relative abundance of *Coriobacteriales* was higher. This order is reduced in colitic mice [45] and enriched in acute liver failure [46]. By contrast, *Coriobacteriales* are more abundant in diseased mucosal ileum tissues from Crohn's disease patients compared to control mucosa from non-IBD patients [47]. *Coriobacteriales* are also more abundant in fecal microbiota of multiple sclerosis patients [48]. Moreover, female mice exposed in utero to air pollution presented higher abundance of *Bacteroidales*. The *Bacteroidales* (or *S24-7* family) are numerically dominant intestinal organisms that associate with the mucosal surface and have properties that both positively and negatively affect the host. The *Bacteroidales* showed a higher abundance in dextran sulfate sodium-induced mice [45, 49]. In adult mice, bacteria from the order *Bacteroidales* are sufficient to promote appearance of intraepithelial lymphocytes in the colon, which are important for the maintenance of a healthy intestinal barrier [50]. In humans, *Bacteroidales* produce bacteroidetocins, a family of broad-spectrum peptide toxins that kill members of the *Bacteroidetes* phylum, including *Bacteroides*, *Parabacteroides*, and *Prevotella* gut species, as well as pathogenic *Prevotella* species [51]. A decrease in adherent *Bacteroidales* diversity (i.e., the number of different *Bacteroides* species per biopsy) has been found at sites with increased inflammation in IBD subjects [52]. Therefore, current knowledge shows that bacteria of the orders *Bacteroidales* and *Coriobacteriales* have a role in the regulation of intestinal homeostasis and are deregulated in certain pathological conditions, but this does not allow us to predict the consequences of their deregulation in females having been exposed in utero to air pollution.

Most of the effects that we observed in the gut as consequences of maternal air pollution exposure are sex-specific. This gender dependency is a classical feature of air pollution impact. For instance, prenatal air pollution exposure has been shown to “program” offspring for increased susceptibility to diet‐induced weight gain and neuroinflammation in adulthood in a sex‐specific manner [53]. Moreover, the effects induced on the immune system of offspring by maternal exposure to diesel exhaust particles (DEP) are also sex-dependent. Indeed, exposure to DEP in utero decreased the frequency of CD1d^high^CD5^+^ B cells in female mice and IFN-γ production by splenocytes in both sexes. Male mice had elevations in macrophage and lymphocyte numbers in response to DEP whereas female mice only had elevated IL-6, MCP-1, and MIP-2 levels [54]. Epidemiological studies have also highlighted gender-specific effects of air pollution on respiratory health. Overall, studies of children suggest stronger effects among boys in early life and among girls in later childhood [55].

Despite accumulating evidence of sex-dependent adverse effects of maternal air pollution exposure, the mechanisms involved are only now starting to be identified. Firstly, prenatal air pollution exposure induces epigenetic modifications in placenta and in cord blood. Notably, ambient PM causes significant epigenomic changes, including alterations in DNA methylation, miRNA regulation, and histone modifications. Birth cohort studies have shown that PM_2.5_ exposure during the last trimester of pregnancy was positively associated with placental methylation of the promoter regions of regulatory genes in the circadian pathway and key DNA repair genes [56]. PM_10_ exposure during the first 2 trimesters of pregnancy was positively associated with placental methylation of HSD11B2 (i.e., genes involved in the glucocorticoid metabolism and fetal growth). Moreover, specific PM_2.5_ pollution exposure windows were associated with altered placental miR-20a, miR-21, miR-146a, and miR-222 expression [57]. Also, prenatal PM_2.5_ exposure was positively associated with cord plasma histone H3 modifications [58]. This altered biomolecular functioning of the placenta may contribute to early and even later-life health consequences.

It is also worth mentioning that others mechanisms, themselves potentially mediated by the epigenetic modifications described above, could also contribute to the gender dependency of air pollution impact. Placental structure and function, as well as feto-placental transcriptome are known to be different between males and females [59]. It has been hypothesised that these differences led to sex-dependent fetus adaptation of in utero environment: males would be less adaptable to shifts in the in utero environment, which then places them at a greater risk for intrauterine morbidities or mortality. Comparatively, females would be more adaptable to changes in the in utero environment at the cost of growth, which may reduce their risk of poor perinatal outcomes. In our study, the in utero pollution was not toxic enough to cause a body weight loss, but the sex-specific differences of feto-placental adaptability were likely to be involved in the gender dependency of the effects observed on the intestinal tract.

Moreover, sex-specific effects affecting other organs could also contribute to the gender dependency of intestinal tract response, through disruption of the critical physiological interconnexion between the gut and these organs. Indeed, it has been reported that, following murine in utero exposure to carbon black particles, liver of male offspring showed selective altered expression in genes belonging to inflammatory, respiratory and nutritional diseases, and genes in female offspring liver were associated mainly with metabolic disease and endocrine systems disorders at postnatal day 2 [58]. Such sex-specific liver disturbances could affect the intestinal tract through impairments of the gut-liver axis [60]. Furthermore, rat prenatal exposure to DEP has been shown to alter microglial development in a more pronounced manner in males than in females [61], which paves the way to possible disturbances of bidirectional gut–brain interactions [62]. Similarly, fetal exposure to air pollution induce lung developmental defects [63], which could impact the key gut-lung axis [64].

## Conclusions

In mice, postnatal exposure to simulated Beijing-like air pollution induced sex-specific effects on intestinal proliferation, maturation, permeability, immune response, and microbiota composition (Additional file 1: Fig. S5). According to the “Developmental Origins of Health and Disease” (DOHaD) concept, early-life alterations induced by intrauterine pollutant exposure may have long-term effects influencing offspring susceptibility to diseases later in adulthood. Therefore, precocious disturbances that we observed in intestinal development following prenatal exposure to air pollution may underlie a higher susceptibility for developing diseases later in life.

## Methods

### Generation of complex air pollution

The innovative approach developed for this study was to realistically simulate the atmospheric mixture in its whole complexity, while maintaining the ability to control, reproduce, and fully characterize the experimental conditions. The CESAM chamber (described at https://cesam.cnrs.fr/; a 4.2 m^3^ stainless steel atmospheric simulation chamber; evaceable down to a few 10^–7^ atm; temperature controlled between + 15 °C and + 60 °C) was used to study the myriad of products arising from the atmospheric oxidation of primary organic compounds. The experimental protocol included a continuous injection of relevant mixtures of primary pollutants (mainly nitrogen oxides, organic compounds from a representative mix of anthropogenic emissions, sulphur dioxide, soot, inorganic salts, and mineral dust particles from the Gobi Desert to simulate a Beijing-like atmosphere from 2017) at low concentrations (ppb levels) in air with the CESAM simulation chamber operating as a slow flow reactor (Additional file 1: Fig. S6). The residence time of simulated air parcels in the experimental volume was fixed to 4 h in order to represent air masses of regional scale. During this time the synthetic mixture was exposed to artificial solar irradiation, allowing secondary pollutants such as O_3_, nitric acid, formaldehyde, peroxyacetyl nitrates (PANs) as well as complex polyfunctional organics including secondary organic aerosols, to be produced and to reach their chemical steady state. Mice were exposed to constant flows of this mixture (Additional file 1: Fig. S1).

### Simulated atmospheric environment at the laboratory

The 2017 China smog event was an extreme air pollution episode that affected East China, initiated by slow air carrying industrial emissions and meteorological conditions, that took place in December of that year. High levels of PM_2.5_ (generated from coal combustion and industrial sources) and low visibility were observed, leading to the closure of airports, highways, and schools [21, 22, 65]. For the simulation of a Beijing-like atmosphere, VOCs were introduced into the atmospheric chamber as precursors, which were the most abundant and commonly emitted particulates in the urban atmosphere of Beijing at the time: isopentane (alkane), propene (alkene), acetylene (alkyne), acetaldehyde (aldehyde), benzene, toluene, and m,p-xylene (aromatic hydrocarbons). The concentrations were determined in order to have stable levels of pollutants in the chamber, and representative of the pollution episode [30]. By the end of 20 h of VOC injection, nitrogen monoxide (NO) was introduced in the chamber and then the lights were turned on, simulating the sunlight irradiation in the troposphere. Additionally, seeds of ammonium sulfate particles were injected in the chamber. They formed contact surfaces for the condensation of oxidized products resulting in the formation of secondary organic aerosols. These particles were produced from the nebulization of an ammonium sulfate solution through an atomizer. In order to realistically simulate an atmosphere representative of Beijing, soot particles were generated using a soot generator (miniCAST Series 5200, which produces combustion soot particles by using a well-defined flame that simulates the combustion in modern combustion engines), based on the combustion of propane. These particles were transferred into a smaller chamber (soot reservoir chamber) and were injected once a day into the CESAM chamber. Moreover, also within 1 h once a day, mineral dust particles were injected, produced through a shaking process from a Gobi Desert sample, into the chamber (simulating a desert storm that impacted Beijing with a mineral dust plume). The maximum PM concentration during the experimental campaign was 200 µg/m^3^ (Additional file 1: Fig. S7).

The atmosphere was monitored by several instruments, including a NOx analyser (chemiluminescence, Horiba APNA370) coupled with a NO_2_ analyser (Teledyne T500U), an O_3_ analyser (UV spectroscopy, Horiba APOA370), a SO_2_ analyser (Horiba APSA370), an in-situ FTIR (Bruker Tensor 37), and for the particulate matter a SMPS (TSI 3080 + TSI3025), a Light-scattering aerosol (Palas Welas Digital 2000) and an Aerosol Chemical Speciation Monitor (Aerodyne) for aerosol speciation.

Table 1 gives an overview of the pollutants defined as reference to qualify a “Beijing-like” atmosphere simulation. This shows how the atmospheric environment to which the mice were exposed is complex (tens of pollutants, both in aerosol and gaseous phases), and representative of the urban situation of Beijing in December 2017 (most of the reference pollutants are present in a relative abundance that is similar to the targeted range) [21, 22].

**Table 1:** Expected and observed ranges of December 2017 “Beijing-like” atmospheric pollutants

Pollutants or components	Observed mean concentration
*Gaseous pollutants*	
Ozone—O_3_ (ppb)	6.34 ± 3.59
Nitrogen monoxide—NO (ppb)	2.98 ± 2.19
Nitrogen dioxide—NO_2_ (ppb)	10.33 ± 4.56
Sulfur dioxide—SO_2_ (ppb)	5.11 ± 2.65
*Particulate pollutants*	
PM_2.5_	
Particle number concentration (part/cm^3^)	2000–10,000
Particle mass concentration (µg/m^3^)	105.46 ± 50.34
Aerosol chemical composition indicators	
Sulfates (µg/m^3^)	5–8
Nitrates (µg/m^3^)	5–10
Ammonium (µg/m^3^)	3–7
Organic carbon (OC) (µg/m^3^)	15–25
Black carbon (BC) (µg/m^3^)	3–5
Gaseous pollutants	
Formaldehyde—HCHO	Identified by PTR-MS
Acetaldehyde—CH_3_CHO	Identified by PTR-MS
Acetone—C_3_H_6_O	Identified by PTR-MS
Methylglyoxal or 2-oxopropanal—C_3_H_4_O_2_	Identified by PTR-MS
Glyoxal or ethanedial—CHOCHO	Identified by PTR-MS

### Experimental design

Forty pregnant C57/BL6J mice arrived from Janvier Labs at GD9. They were directly settled in cages (32.5, 18, 13 cm (LxlxH),TECNIPLAST, France) containing bedding (ASPEN, France) and nesting materials (D00019 lab nest IRR 10KGY and MouseSmartHome + Playtunnelmed shelter, SERLAB, France) as well as shredded filter papers (D000000000020 sizzle pad 8G, UNIQUSE, France). Each cage hosted 5 mice and each compartment (Control air or Pollution) of the cabinet accommodates 4 cages (20 pregnant mice per exposure condition). All the pregnant mice had free access to diet (SSNIFF, Germany Ref S9343-S403) and sterile tap water. In both compartments, the ambient temperature was controlled between 22 and 25 °C, with a humidity from 30 to 50% and a 12 h/12 h light/dark cycle. Surveillance cameras were installed inside the cabinet to observe the behavior and check the status of the mice during the whole exposure period (whole body).

Exposure to air pollution occurred for 7 consecutive days, from GD9 to GD16. At the end of the exposure period, mice were transferred to the animal facility and placed individually into new cages, containing a shelter for mice (SERLAB, France), shredded filter paper (UNIQUSE, France) and nesting material (SERLAB, France). Free access to diet (SSNIFF, Germany Ref S9343-S403) and tap water were also provided to each cage. The delivery occurred between GD19 and GD21. Male and female offspring was sacrificed on the 17th day of life. One male and one female per cage were chosen randomly among the offspring. For each study and each condition, a total of 10 males and 10 females were chosen Intestinal tissues were sampled for histological and molecular biology analyses. The study was carried out in accordance with INSERM recommendations on the treatment of animals and was approved by the ethics committee of the University of Paris-Est Créteil Val de Marne (APAFiS N°: 2017113013439718; N° of Notice: 12/12 / 17-7).

### Histological analysis and image processing

Proximal colon and ileum were fixed in 4% paraformaldehyde overnight, processed, and embedded in paraffin wax by an automatic sample preparation system (LOGOS One, Milestone). Serial histological sections of 4 μm thickness were cut, deparaffinized, rehydrated, and stained with MGG (Carlo Erba reagents, Val de Reuil, France, ref. E460583 and E453612). Images were acquired with a DM5500B microscope (Leica Microsystems, Nanterre, France) and intestinal layers were photographed at a magnification of 20x. Histomorphometric analyses were performed using Image J software. Submucosal cellularity and mucosal surface area in the colon were measured, as well as villus height and crypt depth in the ileum. At least 100 well-oriented mucosa, villi, and crypts were measured in at least 5 individual mice from each group. For vacuole morphologic analyses, an image processing algorithm developed by Lustig et al. was adapted [66]. This algorithm converts a microscope image from a red–green–blue image to a grayscale image: the vacuoles are colored white and all other components are blackened. Based on the overlay of the binary image and the grayscale-processed image, we selected clearly visible vacuoles and Image J allowed the measurement of 1) vacuole number; 2) vacuole area, automatically calculated in pixels and converted to µm^2^; 3) vacuole circularity, a measurement of how closely the shape of the vacuoles approaches that of a circle (circularity can be valued between 0 and 1 inclusively, where 1 is the circularity value of an ideal circle); and 4) cell eccentricity, a measurement of how close the shape of the marked region approaches that of a line or a circle (eccentricity varies between 0 and 1 inclusively, where 0 is the eccentricity value of an ideal circle shape and 1 is the eccentricity value of a line segment).

### Immunohistochemical PCNA staining and quantification

Serial histological sections of 4 µm thickness were cut, deparaffinized, and rehydrated. For antigen unmasking, sections were placed in 10 mM sodium citrate buffer (pH 6.0) and incubated in a heat-induced antigen retrieval chamber for 20 min at 121 °C. After washing, sections were blocked for 30 min with 5% bovine serum albumin in PBS. PCNA primary antibody (NB300-524, Novus Biologicals) was then incubated overnight at 4 °C at a 1:1000 concentration. After washing, tissue sections were incubated for 1 h at room temperature with an Alexa fluor 647-conjugated anti-rabbit PCNA antibody (Thermo Fisher Scientific). Nuclear staining with Hoechst was performed before adding a fluorescent mounting medium. Microscopy was performed using a Leica DM5500 B microscope and data was processed with Leica LAS V3.8 software. Cells positive for PCNA were counted blindly by 2 investigators (5 crypts/slide, 1 slide/animal).

### Quantitative reverse transcription-PCR

Small intestine, cecum, and colon tissue samples were homogenized with ceramic beads using Precellys lysing equipment (Bertin Technologies). Total RNA was extracted with the NucleoSpin RNA kit (Macherey–Nagel). Transcript levels of genes were quantified with the StepOne™ Real-Time PCR System using a SYBR Green PCR master mix (Thermo Fisher Scientific). The primer sequences were designed using Primer Express 3 and are available upon request. Melting curve analyses were performed for each sample and gene. The relative expression of each target gene was normalized to the relative expression of the *Polr2a* housekeeping gene. Quantification of target gene expression was based on the comparative cycle threshold (Ct) value. Fold changes in target genes were analyzed by the 2^−ΔΔCt^ method.

### Western blot

Proteins were extracted from male ileum samples and homogenized in TRI Reagent® lysis buffer (Sigma-Aldrich) according to the manufacturer’s instructions. Twenty micrograms of total protein lysate was separated by SDS-PAGE and electroblotted on nitrocellulose membranes using an Amersham Semi Dry Transfer Unit at 0.8 mA/cm^2^ (Amersham Pharmacia Biotech). Membranes were blocked for 1 h in StartingBlock™ T20 (TBS) with 5% milk blocking buffer (Thermo Fisher Scientific) at room temperature. Then membranes were incubated overnight at 4 °C with 1:500 of rabbit anti-ZO-1 (Thermo Fisher, 61–7300) and 1 h at room temperature with 1:10,000 of mouse anti-* β*-actin (Sigma, A1978). Immunoreactivity was visualized with SuperSignal™ West Pico Plus Chemiluminescent HRP Substrate (Thermo Fisher Scientific). Images were taken using the iBright FL1500 Imaging System (Thermo Fisher Scientific). For relative quantification, the volume intensity of the bands was obtained using iBright software.

### Bacterial DNA extraction and Illumina MiSeq sequencing

Genomic DNA was extracted from the colon luminal content using a DNA stool kit (Macherey Nagel, France). The quantity and purity of DNA (expressed as the ratio of absorbance at 260 nm and 280 nm (A260/A280)) were assessed using a Nanodrop® spectrophotometer. The sequencing library was generated by amplifying the V3-V4 region of the bacterial 16S-rRNA gene using 16S rRNA amplicon generation for MiSeq with the primers Bact-0341 (CCTACGGGNGGCWGCAG) and Bact-0785 (GACTACHVGGGTATCTAATCC). Individual samples were barcoded, pooled to construct the sequencing library, and sequenced using an Illumina MiSeq (Illumina, San Diego, CA) to generate paired-end 2 × 300 bp reads.

### Analysis of sequencing data

Bioinformatic analyses were performed using the QIIME2 pipeline (version 2020.2) [67]. The Divisive Amplicon Denoising Algorithm plug-in (DADA-2) in QIIME2 was used to filter, dereplicate, identify chimeric sequences, and merge PE reads to obtain a set of ASVs for each sample [68]. Then the representative sequences were picked for each ASV. The classify-sklearn plug-in in QIIME2, with the Silva database (version 132), was applied to assign a taxonomic annotation to each representative ASV sequence. In the next step, ASVs identified as eukaryotic contamination (3 ASVs; 12 reads) and external contamination, identified with the decontam package (3 ASVs; 3119 reads), were filtered out [69]. The diversity metrics (alpha and beta diversity) were obtained with the QIIME2 core-metrics-phylogenetic plug-in, with p-sampling depth parameter fixed to 13,781 reads which corresponded to the total frequency that each sample should be rarefied to prior to computing diversity metrics. This sampling depth allowed retention of more than 61% of reads and only one sample was discarded. Tests for differential relative abundance were performed with corncob at the order, family, and genus levels [70].

### Statistics

Results are expressed as mean ± standard error of the mean. Except for metagenomic data, the statistical significance of differences between experimental groups was calculated using the Mann–Whitney nonparametric U test (GraphPad Prism software, USA). Statistical significance was defined as *p* < 0.05. For all experiments, *p < 0.05, **p < 0.01, ***p < 0.005, and = ****p < 0.001.

## Supplementary Information

**Additional file 1: Fig. S1.** Overview of mice exposure in a dedicated exposure device. A realistic atmosphere, representative of a 2017 pollution event in Beijing, was generated in the CESAM atmospheric simulation chamber, at the extreme left of the figure. Mice were exposed to simulated Beijing-like air pollution (both gaseous and particulate phases) directly transferred from CESAM into the top part of the exposure device, while control mice (at the bottom part of the exposure device) were exposed to filtered Beijing-like air pollution during the same period. **Fig. S2.** Dam and pup general outcomes. A Litter size. B Offspring gender proportion. C Dam body weight. D Offspring body weight. **Fig. S3.** Enteroendocrine cell populations markers in female ileum. Transcript levels of *Tac1*, *Gcg*, *Pyy*, *Gip*, *Nts*, and *Sct* in mice exposed to control air or Beijing-like air (n = 10/group). **Fig. S4.** Evenness and Simpson α-diversity index of luminal content microbiota in male and female mice exposed in utero to control air or Beijing-like air (n = 10/group). **Fig. S5.** Table summarizing the endpoints studied and the significant differences observed. **Fig. S6.** Numerical modelling of the chemical activity inside CESAM chamber. **Fig. S7.** Concentration of PM (µg/m^3^) during the experimental campaign. Each day a maximum peak is resulting from injection of soot and dust particles.

## Data Availability

The datasets used and/or analyzed during the current study are available from the corresponding author on reasonable request.
