# Supplementary material for: Murine in utero exposure to simulated complex urban air pollution disturbs offspring gut maturation and microbiota during intestinal suckling-to-weaning transition in a sex-dependent manner
Source: Part Fibre Toxicol. 2022 Jun 15;19:41. doi: 10.1186/s12989-022-00481-y (PMC9199156; doi:10.1186/s12989-022-00481-y)
Supplement: Supplementary file 1 — Additional file 1: Fig. S1. Overview of mice exposure in a dedicated exposure device. A realistic atmosphere, representative of a 2017 pollution event in Beijing, was generated in the CESAM atmospheric simulation chamber, at the extreme left of the figure. Mice were exposed to simulated Beijing-like air pollution (both gaseous and particulate phases) directly transferred from CESAM into the top part of the exposure device, while control mice (at the bottom part of the exposure device) were exposed to filtered Beijing-like air pollution during the same period. Fig. S2. Dam and pup general outcomes. A Litter size. B Offspring gender proportion. C Dam body weight. D Offspring body weight. Fig. S3. Enteroendocrine cell populations markers in female ileum. Transcript levels of Tac1, Gcg, Pyy, Gip, Nts, and Sct in mice exposed to control air or Beijing-like air (n = 10/group). Fig. S4. Evenness and Simpson α-diversity index of luminal content microbiota in male and female mice exposed in utero to control air or Beijing-like air (n = 10/group). Fig. S5. Table summarizing the endpoints studied and the significant differences observed. Fig. S6. Numerical modelling of the chemical activity inside CESAM chamber. Fig. S7. Concentration of PM (µg/m3) during the experimental campaign. Each day a maximum peak is resulting from injection of soot and dust particles. [file 12989_2022_481_MOESM1_ESM.docx]

**Additional file 1**

**Murine *in utero* exposure to simulated complex urban air pollution disturbs intestinal suckling-to-weaning transition in mice in a sex-dependent manner.**

Eva Guilloteau^1^, Patrice Coll^2^, Zhuyi Lu^3^, Madjid Djouina^1^, Mathieu Cazaunau^4^, Christophe Waxin^1^, Antonin Bergé^2^, Ségolène Caboche^5^, Aline Gratien^2^, Elie Al Marj^2^, David Hot^5^, Laurent Dubuquoy^1^, David Launay^1^, Cécile Vignal^1^, Sophie Lanone^3^, Mathilde Body-Malapel^1^

1. *Univ. Lille, INSERM, CHU Lille, U1286 - INFINITE - Institute for Translational Research in Inflammation, F-59000 Lille, France*
2. ***Université Paris Cité and Univ Paris Est Créteil, CNRS, LISA, F-75013 Paris, France***
3. *Univ. Paris Est Créteil, INSERM, IMRB, F-94010 Créteil, France*
4. *Univ. Paris Est Créteil and Université Paris Cité, CNRS, LISA, F-94010 Créteil, France*
5. *Univ. Lille, CNRS, INSERM, CHU Lille, Institut Pasteur de Lille, UMR2014-US41-PLBS-Plateformes Lilloises de Biologie & Santé, F-59000, Lille, France*


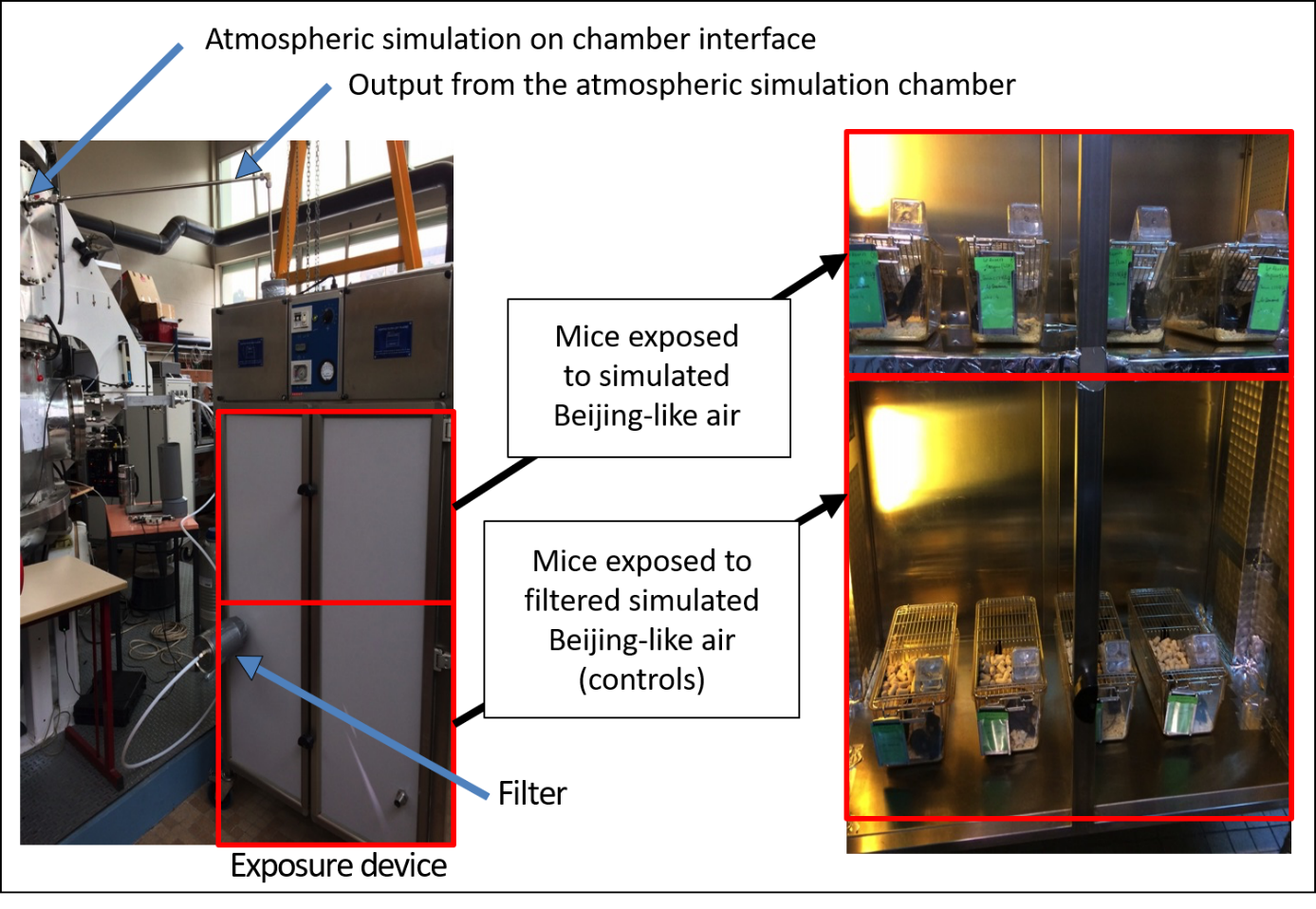


**Fig S1** Overview of mice exposure in a dedicated exposure device. A realistic atmosphere, representative of a 2017 pollution event in Beijing, was generated in the CESAM atmospheric simulation chamber, at the extreme left of the figure. Mice were exposed to simulated Beijing-like air pollution (both gaseous and particulate phases) directly transferred from CESAM into the top part of the exposure device, while control mice (at the bottom part of the exposure device) were exposed to filtered Beijing-like air pollution during the same period.

**
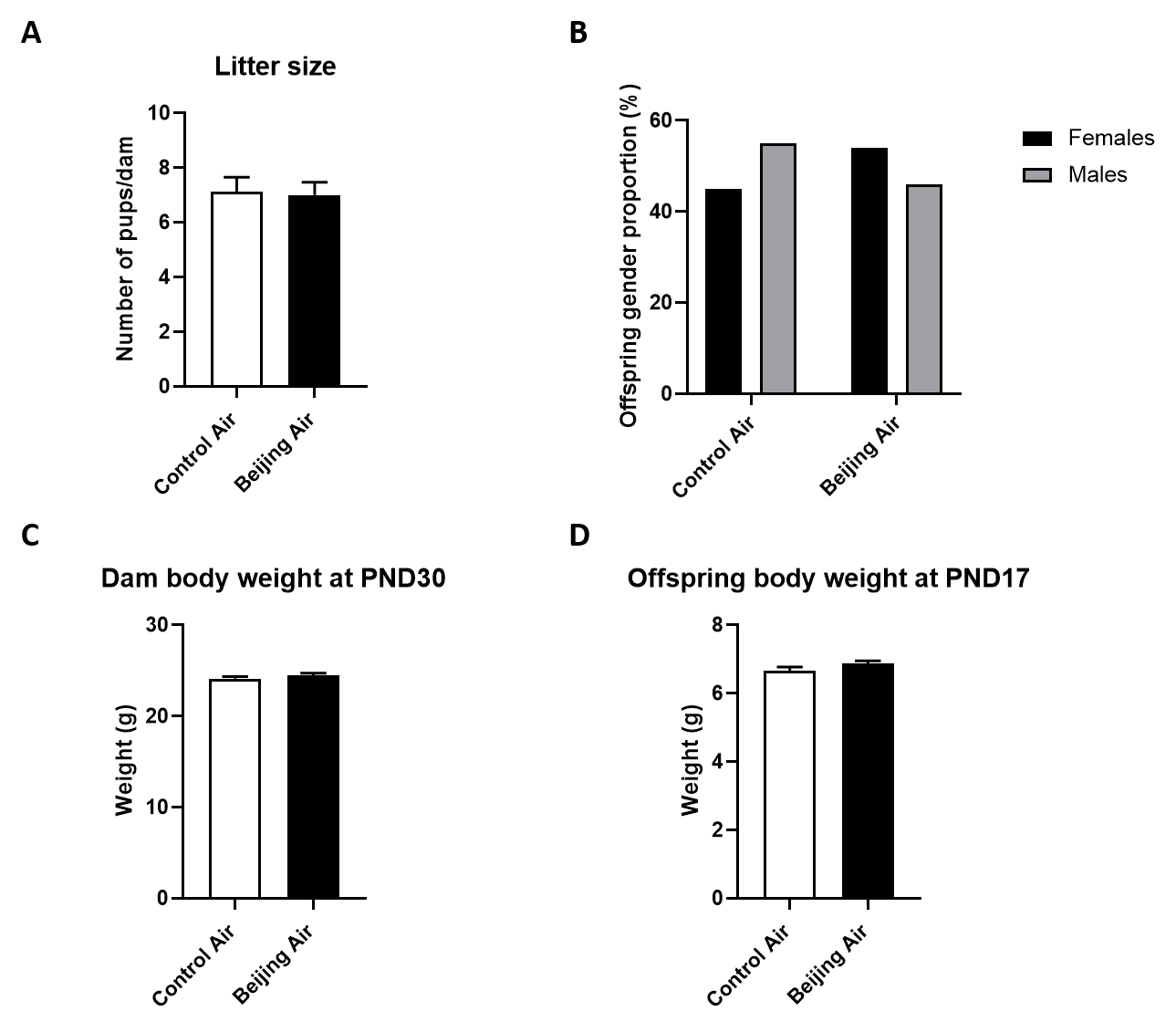
****Fig S2** Dam and pup general outcomes. **A** Litter size. **B** Offspring gender proportion. **C** Dam body weight. **D** Offspring body weight.


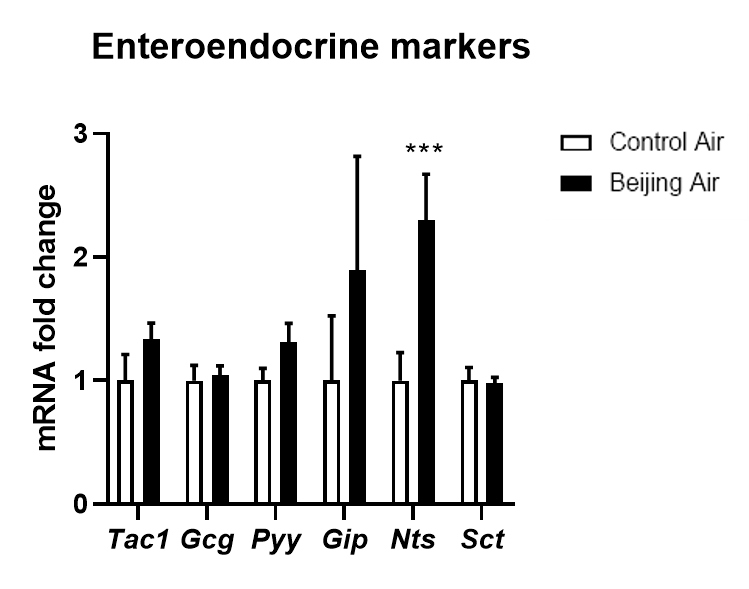


**Fig S3** Enteroendocrine cell populations markers in female ileum. Transcript levels of *Tac1*, *Gcg*, *Pyy*, *Gip*, *Nts*, and *Sct* in mice exposed to control air or Beijing-like air (n=10/group).

**
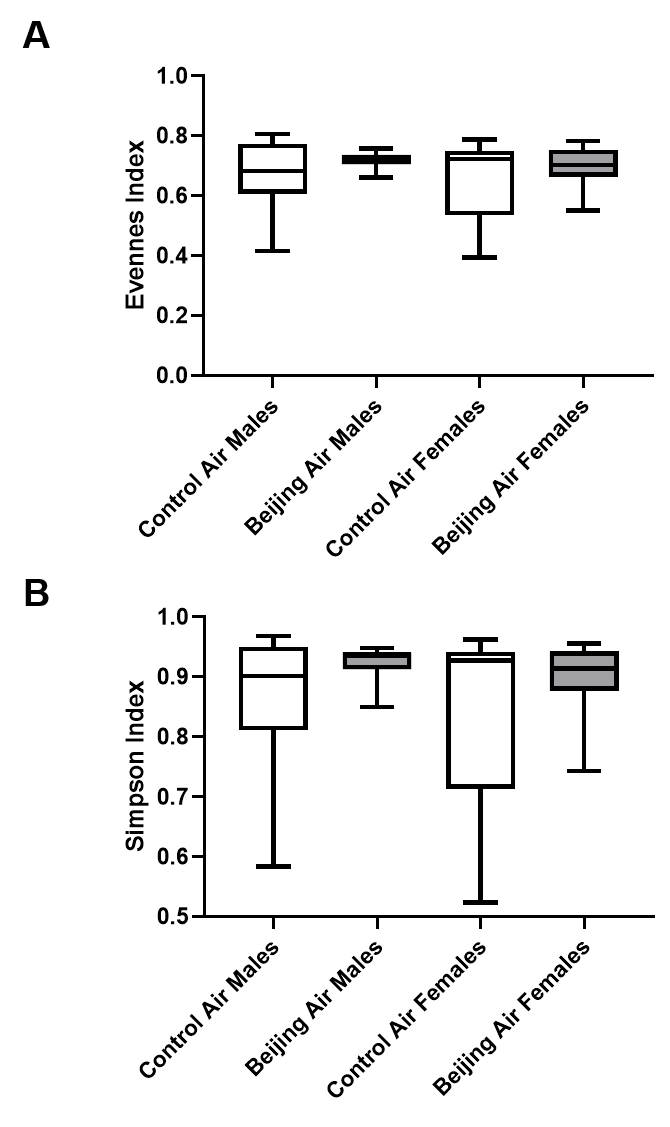
**

**Fig S4** Evenness and Simpson α-diversity index of luminal content microbiota in male and female mice exposed *in utero* to control air or Beijing-like air (n=10/group).

| Maturation/differentiation | | Male | Female |
| --- | --- | --- | --- |
| Proximal colon | Submucosal cellularity |  |  |
|  | Mucosal surface area |  |  |
| Ileum | Villus length | ↘** |  |
|  | Crypt depth | ↘* |  |
|  | Villus/Crypt ratio |  |  |
|  | Vacuole number per villus |  |  |
|  | Mean vacuolated area per villus | ↘**** |  |
|  | Vacuole index | ↘*** |  |
|  | Mean eccentricity index | ↘*** |  |
|  |  |  |  |
| Proximal colon | PCNA positive cells/crypt |  |  |
|  | Lgr5 mRNA |  | ↘** |
|  | Alpi mRNA | ↗* |  |
|  | Epcam mRNA | ↗*** |  |
|  | Car2 mRNA | ↗** |  |
|  | Muc2 mRNA | ↘* |  |
|  | Lyz1 mRNA |  |  |
|  | ChrgA mRNA |  |  |
|  | Pou2f3 mRNA |  |  |
|  | Ass1 mRNA | ↘*** |  |
|  | Fcgrt mRNA | ↘* |  |
|  | Prdm1 mRNA |  |  |
|  | Lct mRNA | ↘* |  |
|  | Treh mRNA |  |  |
|  | Arg2 mRNA | ↘* | ↘* |
|  | Sis mRNA | ↗* |  |
| Ileum | PCNA positive cells/crypt | ↗*** |  |
|  | Lgr5 mRNA |  |  |
|  | Alpi mRNA |  |  |
|  | Epcam mRNA |  | ↗* |
|  | Car2 mRNA |  | ↗* |
|  | Muc2 mRNA |  |  |
|  | Lyz1 mRNA |  |  |
|  | ChrgA mRNA |  | ↗** |
|  | Pou2f3 mRNA |  |  |
|  | Ass1 mRNA |  |  |
|  | Fcgrt mRNA |  |  |
|  | Prdm1 mRNA |  |  |
|  | Lct mRNA |  |  |
|  | Treh mRNA |  |  |
|  | Arg2 mRNA |  |  |
|  | Sis mRNA |  | ↗* |
|  |  |  |  |
| Inflammation |  | Male | Female |
| Proximal colon | Tbx21 mRNA |  |  |
|  | Tnfa mRNA |  |  |
|  | Ifng mRNA |  |  |
|  | Gata3 mRNA |  |  |
|  | Il4 mRNA |  |  |
|  | Il5 mRNA |  |  |
|  | Il13 mRNA |  | ↗** |
|  | Rorc mRNA |  |  |
|  | IL17a mRNA |  |  |
|  | Il22 mRNA |  |  |
|  | Foxp3 mRNA |  |  |
|  | Il10 mRNA | ↗*** | ↗** |
|  | Tgfb mRNA |  |  |
| Cecum | Tbx21 mRNA |  |  |
|  | Tnfa mRNA |  |  |
|  | Ifng mRNA |  |  |
|  | Gata3 mRNA |  |  |
|  | Il4 mRNA | ↗* | ↗** |
|  | Il5 mRNA | ↗* |  |
|  | Il13 mRNA |  |  |
|  | Rorc mRNA | ↗*** |  |
|  | IL17a mRNA | ↘* |  |
|  | Il22 mRNA | ↘** |  |
|  | Foxp3 mRNA |  |  |
|  | Il10 mRNA |  |  |
|  | Tgfb mRNA |  |  |
| Ileum | Tbx21 mRNA |  |  |
|  | Tnfa mRNA |  |  |
|  | Ifng mRNA |  |  |
|  | Gata3 mRNA |  |  |
|  | Il4 mRNA |  |  |
|  | Il5 mRNA |  |  |
|  | Il13 mRNA |  |  |
|  | Rorc mRNA | ↗* |  |
|  | IL17a mRNA |  |  |
|  | Il22 mRNA |  |  |
|  | Foxp3 mRNA |  |  |
|  | Il10 mRNA |  |  |
|  | Tgfb mRNA |  |  |
|  |  |  |  |
| Permeability markers | | Male | Female |
| Proximal colon | Ocln mRNA |  |  |
|  | Tjp1 mRNA |  |  |
|  | Cldn4 mRNA |  | ↗* |
| Cecum | Ocln mRNA | ↗** |  |
|  | Tjp1 mRNA | ↘* | ↗* |
|  | Cldn4 mRNA | ↘** |  |
| Ileum | Ocln mRNA |  |  |
|  | Tjp1 mRNA | ↘** |  |
|  | Cldn4 mRNA |  |  |
|  | ZO1 (TJP1) protein | ↘* |  |
|  |  |  |  |
| Colon microbiota | | Male | Female |
|  | Chao 1 index |  |  |
|  | Weighted Unifrac index |  | ↘* |
|  | Bacteroidales |  | ↗*** |
|  | Coriobacteriales |  | ↗* |
|  | Firmicutes/Bacteroides ratio |  | ↘** |

**Fig S5** Table summarizing the endpoints studied and the significant differences observed. ↘= significant decrease. ↗= significant increase. * p<0.05, ** p<0.01, *** p<0.005 as determined by the Mann-Whitney U test.


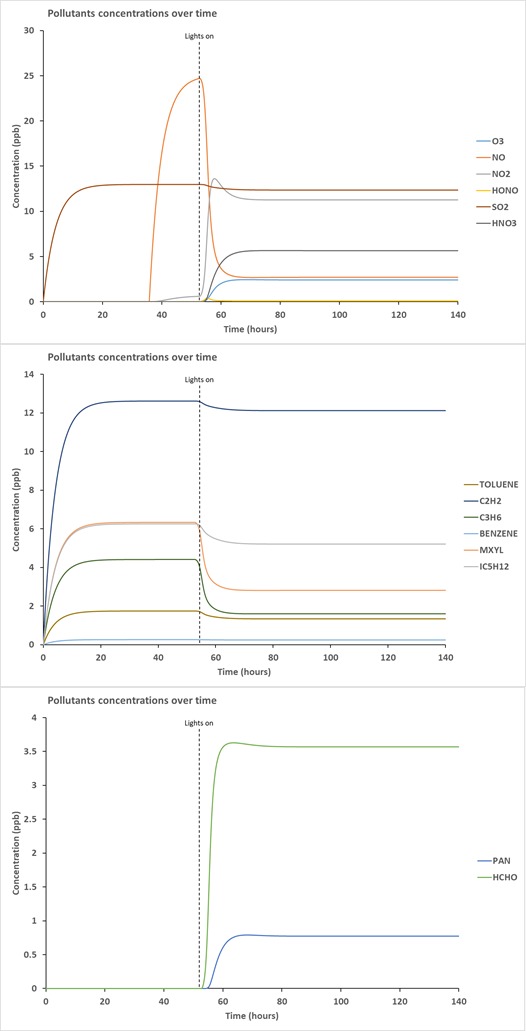


**Fig S6** Numerical modelling of the chemical activity inside CESAM chamber.

**Fig S7** Concentration of PM (µg/m^3^) during the experimental campaign. Each day a maximum peak is resulting from injection of soot and dust particles.
